# Carotenoid Biosynthesis in *Fusarium*

**DOI:** 10.3390/jof3030039

**Published:** 2017-07-07

**Authors:** Javier Avalos, Javier Pardo-Medina, Obdulia Parra-Rivero, Macarena Ruger-Herreros, Roberto Rodríguez-Ortiz, Dámaso Hornero-Méndez, María Carmen Limón

**Affiliations:** 1 Departamento de Genética, Facultad de Biología, Universidad de Sevilla, 41012 Sevilla, Spain; jpardo6@us.es (J.P.-M.); duly@us.es (O.P.-R.); macarenarugerherreros@gmail.com (M.R.-H.); lrrodriguezor@conacyt.mx (R.R.-O.); carmenlimon@us.es (M.C.L.); 2 Present Address: CONACYT-Instituto de Neurobiología-UNAM, Juriquilla, Querétaro 076230, Mexico; 3 Departamento de Fitoquímica de los Alimentos, Instituto de la Grasa, CSIC, Campus Universidad Pablo de Olavide, 41013 Sevilla, Spain; hornero@ig.csic.es

**Keywords:** neurosporaxanthin, xanthophyll, apocarotenoid, retinal, torulene, photoinduction, RING-Finger protein, carotenoid gene cluster

## Abstract

Many fungi of the genus *Fusarium* stand out for the complexity of their secondary metabolism. Individual species may differ in their metabolic capacities, but they usually share the ability to synthesize carotenoids, a family of hydrophobic terpenoid pigments widely distributed in nature. Early studies on carotenoid biosynthesis in *Fusarium* *aquaeductuum* have been recently extended in *Fusarium fujikuroi* and *Fusarium oxysporum*, well-known biotechnological and phytopathogenic models, respectively. The major *Fusarium* carotenoid is neurosporaxanthin, a carboxylic xanthophyll synthesized from geranylgeranyl pyrophosphate through the activity of four enzymes, encoded by the genes *carRA*, *carB*, *carT* and *carD*. These fungi produce also minor amounts of β-carotene, which may be cleaved by the CarX oxygenase to produce retinal, the rhodopsin’s chromophore. The genes needed to produce retinal are organized in a gene cluster with a rhodopsin gene, while other carotenoid genes are not linked. In the investigated *Fusarium* species, the synthesis of carotenoids is induced by light through the transcriptional induction of the structural genes. In some species, deep-pigmented mutants with up-regulated expression of these genes are affected in the regulatory gene *carS*. The molecular mechanisms underlying the control by light and by the CarS protein are currently under investigation.

## 1. Introduction

The genus *Fusarium* is a complex group of phytopathogenic fungi, consisting of more than one thousand species [1], many of which have been the object of detailed attention [2]. Different *Fusarium* species were found to produce a large array of secondary metabolites. Some of them are pigments, and color variations have been used from long ago as distinctive traits of different strains (see, e.g., [3]). A most characteristic class of fungal pigments are the carotenoids, a family of lipophilic terpenoids ubiquitous in all major taxonomic groups [4,5]. The carotenoids are synthesized by all photosynthetic organisms, from cyanobacteria to higher plants, but they are also produced by diverse heterotrophic microorganisms, including fungi [6,7] and non-photosynthetic bacteria [8,9]. There are more than 750 natural carotenoids [5], providing typical yellowish, orange or reddish pigmentations to many plant organs, microorganisms and animals. With some notable exceptions in aphids [10], the animals are generally unable to synthesize carotenoids, and they get them through the diet. Carotenoids are especially relevant in photosynthetic species, where they play essential roles in light harvesting and photoprotection of the photosynthetic machinery [11] and in animals as a source for retinoids, i.e., retinal and retinoic acid [12]. However, the carotenoids do not seem to play essential roles in fungi, where their absence has no apparent phenotypic consequences apart of altered pigmentation [13].

**Figure 1:**
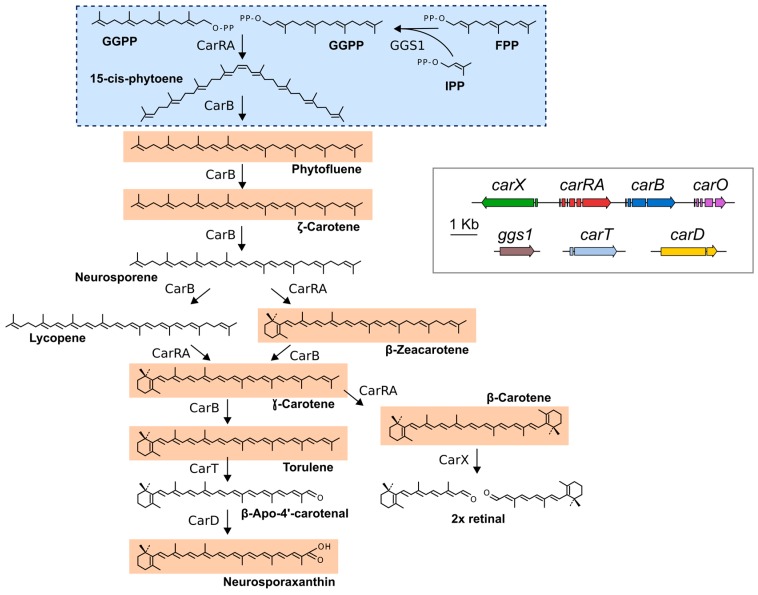
Carotenoid biosynthesis in *Fusarium*. Gene products responsible for the biosynthetic steps are indicated close to each arrow. Genomic organization of the genes is shown in the inserted box. Carotenoids detected in the High Performance Liquid Chromatography (HPLC) analyses described in Figure 4 are shaded in orange. Compounds shaded in blue (dotted box) are not detected in the chromatograms because of lack of absorption in the covered detection range (350–600 nm).

As terpenoids, the synthesis of carotenoids derives from the condensation of C_5_ isoprene units [4,5]. The universal terpenoid precursor is isopentenyl pyrophosphate (IPP), which can be synthesized either from mevalonate, produced from hydroxymethylglutaryl coenzyme A (HMG-CoA) (mevalonate pathway), or from derivatives of d-1-deoxyxylulose 1-phosphate, generated from the condensation of pyruvate and glyceraldehyde 3-phosphate (non-mevalonate pathway, [14]). In the cases investigated, fungal IPP is produced through the mevalonate pathway, while for carotenoids in bacteria and photosynthetic species IPP is produced through the non-mevalonate pathway, localized in plastids in the eukaryotic organisms. The early biosynthetic steps involve the sequential additions of IPP (isoprene, C_5_) units to produce geranyl pyrophosphate (GPP, C_10_), farnesyl pyrophosphate (FPP, C_15_), and geranylgeranyl pyrophosphate (GGPP, C_20_), carried out by prenyl transferases [4,5]. The first molecule with the characteristic aliphatic carotenoid-like structure is the colorless 15-*cis*-phytoene, consisting of a symmetrical polyene chain formed by the condensation of two GGPP units by phytoene synthase (Figure 1). The light-absorbing features of colored carotenoids are due to the presence of a chromophore, consisting of a series of conjugated double bonds generated by the activity of desaturase enzymes. Different subsequent chemical changes, usually the introduction of a cyclic end group (β and ε rings are the most common, while γ, κ, φ and χ rings are less frequent) on at least one of the ends of the molecule, and/or oxidative reactions (e.g., hydroxylation, epoxidation, carboxylation, esterification, etc), gives rise to the large diversity of known carotenoids [5].

## 2. *Fusarium* Carotenoids

Early reports mentioned the occurrence of carotenoids in *Fusarium* species, e.g., *F. oxysporum* [3], but the first detailed biochemical analyses were described in *Fusarium aquaeductuum* [15]. Such analyses showed the occurrence of non-polar carotenoids and an acidic carotenoid fraction, which reminded the one assigned as the xanthophyll neurosporaxanthin (hereafter NX) in *Neurospora crassa* [16]. NX is a carboxylic C_35_ apocarotenoid, chemically defined as β-apo-4′-carotenoic acid [17]. NX is not a frequent carotenoid in fungi, and to date, besides the genera *Neurospora* and *Fusarium*, it has been only found in *Verticillium* [18,19] and *Podospora* [20].

Carotenoid analyses were refined in *F. aquaeductuum* in subsequent years, and confirmed the occurrence of NX together with precursor neutral carotenes, that included ζ-carotene, neurosporene, lycopene, γ-carotene, and torulene [21]. Later studies in *Fusarium fujikuroi*, and data presented in Section 5, corroborated the carotenoid composition of *F. aquaeductuum* with the exception of the detection of β-zeacarotene instead of lycopene. This indicates that the cyclization may be achieved either on neurosporene or on lycopene, depending on the species. Taken together, the chemical analyses are consistent with the biosynthetic pathway depicted in Figure 1. From phytoene, five desaturations and the cyclization of the non-saturated end yield the reddish torulene. The steps from torulene to NX were deduced from the enzymatic activities of the responsible enzymes, discussed below. In *F. fujikuroi* there are minor amounts of β-carotene [22], showing that the CarRA cyclase is also able to recognize γ-carotene as substrate. Because of its carboxylic group, NX may be a subject of esterification. In a marine *Fusarium* species, a fraction of the produced NX is accumulated as a glycosyl ester [23].

## 3. Genes and Enzymes of Carotenoid Metabolism in *Fusarium*

Some of the enzymes responsible for early steps of the terpenoid pathway were first identified in *F. fujikuroi* thanks to the similarity with formerly known enzymes. The typical cloning protocol consisted in the identification of conserved protein segments, the design of degenerate primers from such sequences to clone the corresponding internal gene segment by PCR, and the use of the amplified segment as a probe to clone the whole gene through the screening of a genomic library. Such methodology led to clone the genes for HMG-CoA reductase [24], producing mevalonate, and the prenyl transferases responsible for the synthesis of FPP [25] and its conversion to GGPP [26]. The mutants of these genes are expected to be non-viable because of their participation in the synthesis of essential terpenoids, and therefore their functions were inferred from their close relatedness with orthologs in other organisms. *F. fujikuroi* contains a second GGPP synthase located in the cluster for the synthesis of gibberellins, growth-inducing plant hormones of the terpenoid family. This second GGPP synthase gene is presumably specific for the synthesis of these secondary metabolites, as it indicates its coregulation with most of the genes from the gibberellin cluster [27].

The first gene specifically involved in the synthesis of carotenoids known in *Fusarium* was *carB* [28], identified by a PCR strategy similar to the one mentioned above through its sequence similarity to the *N. crassa* ortholog *al-1* [29]. The function of the gene was confirmed by complementation of a phytoene-accumulating albino mutant and by generation of the same phenotype by *carB* targeted mutation. The CarB enzyme is responsible for the five desaturation steps of the NX biosynthetic pathway, as it indicated the identification of a *carB* mutant allele specifically affected in the fifth desaturation [30]. This was in accordance with former observations with *al-1* of *N. crassa*: its expression in *Escherichia coli* or in vitro studies with purified AL-1 enzyme demonstrated its capacity to carry out the five desaturation steps for NX biosynthesis in this species [31]. Closely linked to *carB* it was found the gene *carRA* (Figure 1), orthologous of phytoene synthase *al-2* of *N. crassa* [32] and interpreted as the phytoene synthase of *Fusarium*, a conclusion consistent with the albino phenotype of their mutants [33]. In both species, a carboxylic domain with similarity to carotene cyclases precedes the phytoene synthase domain. The cyclase domain has a high conservation with that of the orthologous gene *crtYB* of the yeast *Xanthophyllomyces dendrorhous*, where the cyclase activity was biochemically demonstrated [34]. No mutants specifically affected in the cyclase activity have been described in *Fusarium*, but the mutants of the cyclase domain of the *al-2* gene of *N. crassa* were shown to be defective in the cyclization step [35,36]. Taken together, the available data strongly suggest the same function for the amino domain of the CarRA protein of *F. fujikuroi*.

The reactions from torulene to NX include a carotenoid cleavage step, typically achieved by a family of enzymes known as carotenoid oxygenases [37]. A gene for an enzyme of this family, called *carX*, was found to be linked and transcribed divergently from the gene *carRA* (Figure 1). However, its targeted mutation did not impede the synthesis of NX [38], and the study of its enzymatic activity revealed that it cleaved β-carotene symmetrically to produce retinal [39]. Interestingly, the *carX*/*carRA*/*carB* cluster is linked to a rhodopsin gene, called *carO* because of its regulatory connections with the rest of the genes of the cluster (see next sections). The rhodopsins typically bind retinal as a light-absorbing prosthetic group, providing coherence to the gene organization of the cluster: the genes *carRA*, *carB*, and *carX* are necessary to produce retinal, which is presumably used by the rhodopsin encoded by the coregulated *carO* gene. Retinal might be also used for a second rhodopsin, encoded by the gene *opsA* [40]. In addition, retinal might be subject to further chemical reactions. The *Fusarium* genomes contain a gene for an aldehyde dehydrogenase highly similar to RALDH enzymes converting retinal to retinoic acid in mammals. This enzyme, that was called CarY, was investigated in *F. verticillioides* and found to exhibit such enzymatic activity in vitro [41]. Targeted mutation of the *carY* gene in *F. verticillioides* had no effect on carotenogenesis, but resulted in diverse developmental alterations. However, no retinoic acid could be detected in the control fungal cells and hence the biochemical function of the CarY enzyme in *Fusarium* has not been solidly established.

The *Fusarium* genes responsible for the conversion of torulene to NX have been recently described. The search for genes encoding other putative carotenoid oxygenases in the *Fusarium* genome databases led to identify the gene *carT*, whose function was revealed by the presence of a mutation in a torulene accumulating mutant and its genetic complementation with the wild-type *carT* allele [42]. As biochemical confirmation, purified CarT enzyme efficiently cleaved torulene in vitro to produce β-apo-4′-carotenal. As additional support, the targeted mutation of its ortholog in *N. crassa*, called *cao-2* from carotenoid oxygenase, resulted in the block of NX production and the accumulation of torulene [43]. These findings established a new enzymatic class in the carotenoid oxygenase family. The function of the gene *carT* in *Fusarium* carotenogenesis, together with those of the genes *carRA* and *carB*, was also confirmed by targeted gene disruption in *Gibberella zeae*, teleomorph of *Fusarium graminearum* [44].

The product of CarT, β-apo-4′-carotenal, requires a further oxidation to produce NX. The responsible gene was first discovered in *N. crassa* thanks to the study of a yellow mutant, called *ylo-1*. This mutant exhibited a puzzling biochemical phenotype, since it contained a complex carotenoid mixture that varied with the culture conditions and that did not include NX [45,46]. The gene *ylo-1* encodes an aldehyde dehydrogenase mediating the last step of NX biosynthesis, as demonstrated in its ability to complement the *ylo-1* mutation [47] and the capacity of purified YLO-1 protein to convert in vitro β-apo-4′-carotenal into NX. This finding allowed the identification of the orthologous gene in *F. fujikuroi*, that was called *carD* [48]. The function of *carD* was confirmed by targeted mutation, which resulted in the lack of NX and the accumulation of unusual carotenoids, interpreted as β-apo-4′-carotenal derivatives.

## 4. Regulation by Light

Early observations in *F. oxysporum* revealed that illumination promotes the accumulation of carotenoids in the mycelium [3]. First detailed studies on the effect of light were carried out with *F. aquaeductuum*. In an initial report, focused on the effect of temperature on the photoinduction process [49], the time course of the response to a transient light exposure revealed a gradual carotenoid accumulation that reached a maximum at about 12 h after illumination. However, the synthesis keeps increasing at a slower rate for at least three days if the culture is maintained under light [50]. Regarding the effect of temperature, the secondary reaction (formation of colored carotenoids) decreases at lower temperatures while the primary reaction to light, presumably a photochemical process, is independent of temperature in the range of 5–25 °C.

The photoinduction is ineffective under anaerobic conditions, as indicated by the lack of carotenoid accumulation if the illuminated mycelia are transferred to an oxygen-free atmosphere [51]. However, the photoinduced state is maintained, as indicated by the onset of carotenoid biosynthesis if the aerobic conditions are restored, even in the dark. Under aeration, the photoinduction requires protein synthesis, as shown by the lack of response if cycloheximide is added before or immediately after light exposure [50]. As happens with anaerobiosis, the removal of cycloheximide allows the start of carotenoid accumulation at any time in the dark, at least up to 30 h, pointing again to a high stability of the induced state of the photoreception system. In an ingenious experiment, when a *F. aquaeductuum* culture was illuminated, incubated in the dark to allow the start of enzyme production, and then exposed to anaerobiosis and to cycloheximide to block further enzyme production, there was no carotenoid production. However, if the culture was returned afterwards to aerobiosis, carotenoid biosynthesis started even in the presence of cycloheximide, indicating that the effect of anaerobiosis is at the level of enzyme activity, and that the enzymes are sufficiently stable [51].

In *F. aquaeductuum*, the effect of light may be partially replaced by the addition of the sulfhydryl oxidizing reagents p-chloro- and p-hydroxymercuribenzoate [50,52] or the oxidative reagent hydrogen peroxide [53], suggesting that oxidation of -SH groups plays a role in the light detection system. Accordingly, the photoinduction is abolished upon addition of reducing agents as dithionite and hydroxylamine, but the response is recuperated if the reducing agent is removed, indicating the recovery of the photoreceptor system [53]. However, while a short exposure to light is sufficient for a sustained photoinduction, p-hydroxymercuribenzoate must be all the time present to maintain its stimulating effect, and such stimulation is additive with that of light [54], indicating different mechanisms of action. Moreover, p-hydroxymercuribenzoate was ineffective in *F. fujikuroi* in the dark, while this species exhibits a similar photoinduction [55].

The study of light dependence of the photoinduction in *F. aquaeductuum* [56] showed that the amount of produced carotenoids depends on the incident light over a 100-fold range, with a reciprocity law holding true over a wide range of exposure times and light intensities. Action spectrum for NX photoinduction extends from 400 to 500 nm, with maxima at 375/380 nm and 450/455 nm and a shoulder at 430/440 nm, a shape consistent with the participation of a flavin photoreceptor. No induction is detected with wavelengths beyond 500 nm, and actually red light proved to be ineffective, discarding the participation of a phytochrome-like photoreceptor [57]. However, incubation of the fungus with methylene blue or toluidine blue allows it to respond to red light [58], suggesting that these chemicals may act as artificial photoreceptors. The shape of the action spectrum also discards the carotenoids as light-absorbing chromophores. As supporting evidence, the accumulation of phytoene in the albino *carB* mutant SG43 is still dependent on light [22], and the mutants of gene *carX* [38], involved in retinal formation, or those of the rhodopsin genes *carO* [59] and *opsA* [40] exhibit a full carotenoid photoinduction.

In *N. crassa*, a fungus with a similar action spectrum for light-induced carotenoid biosynthesis [60], the photoinduction is totally dependent on the White Collar complex, consisting of the photoreceptor WC-1 and its partner WC-2 (reviewed by [61]). However, the mutants of the *wc-1* orthologous genes of *F. fujikuroi* (*wcoA* [62]) and *F. oxysporum* (*wc1* [63]) conserve to different extents a detectable carotenoid photoinduction under continuous illumination. The targeted mutation of the orthologous *wc-1* and *wc-2* genes in *F. graminearum*, *fgwc-1* and *fgwc-2*, result in a paler pigmentation of the surface colonies under light, but the levels of carotenoids either in the wild type and the mutants were not chemically determined [64].

The analysis of carotenoid accumulation on surface cultures revealed a two-stage response in *F. fujikuroi*, with a first rapid increase dependent on WcoA and a slower subsequent carotenoid accumulation depending on another flavin photoreceptor, the DASH cryptochrome CryD [65]. The photoactivity of this flavin photoreceptor has been experimentally demonstrated [66], and its participation as a second photoreceptor explains the maintenance of photoinduced carotenoid accumulation in the *wcoA* mutants under constant illumination. The photoreceptors WcoA and CryD are not only involved in the regulation of carotenogenesis, as indicated by the alteration in the production of other pigments and secondary metabolites in their corresponding mutants [62,67], even in the dark in the case of WcoA.

**Figure 2:**
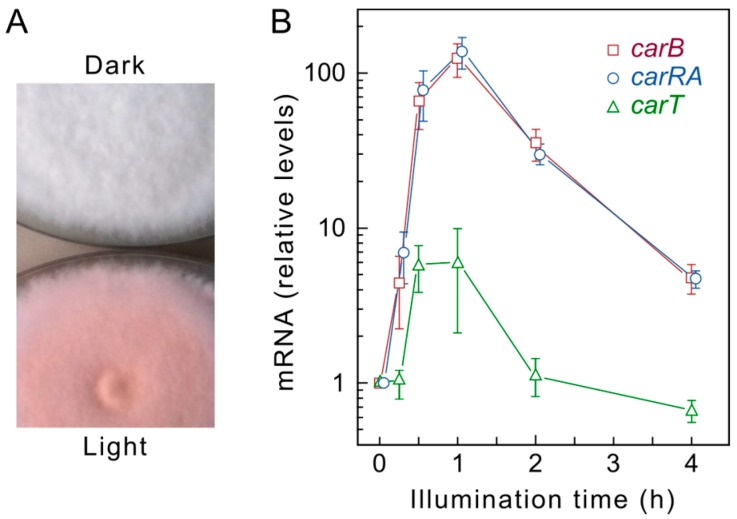
Photoinduction of carotenogenesis in *F. fujikuroi*. (**A**) Aspect of 7-day old colonies of the wild type IMI58289 grown on minimal medium in the dark or under continuous illumination; (**B**) Example of the kinetics of mRNA accumulation for the genes *carRA*, *carB,* and *carT* in the wild type FKMC1995. Transcript levels were determined by RT-qPCR (real time quantitative PCR) and referred to those of β-tubulin gene. Levels in the dark for each gene were taken as 1 (adapted from [65]).

**Figure 3:**
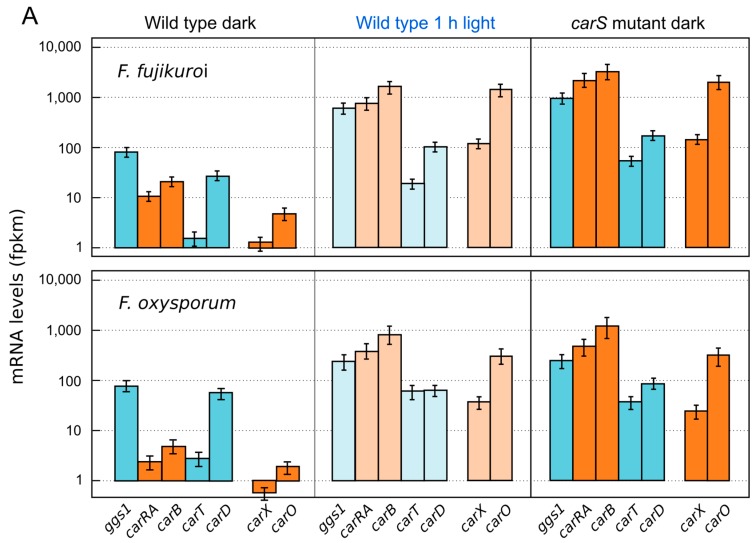

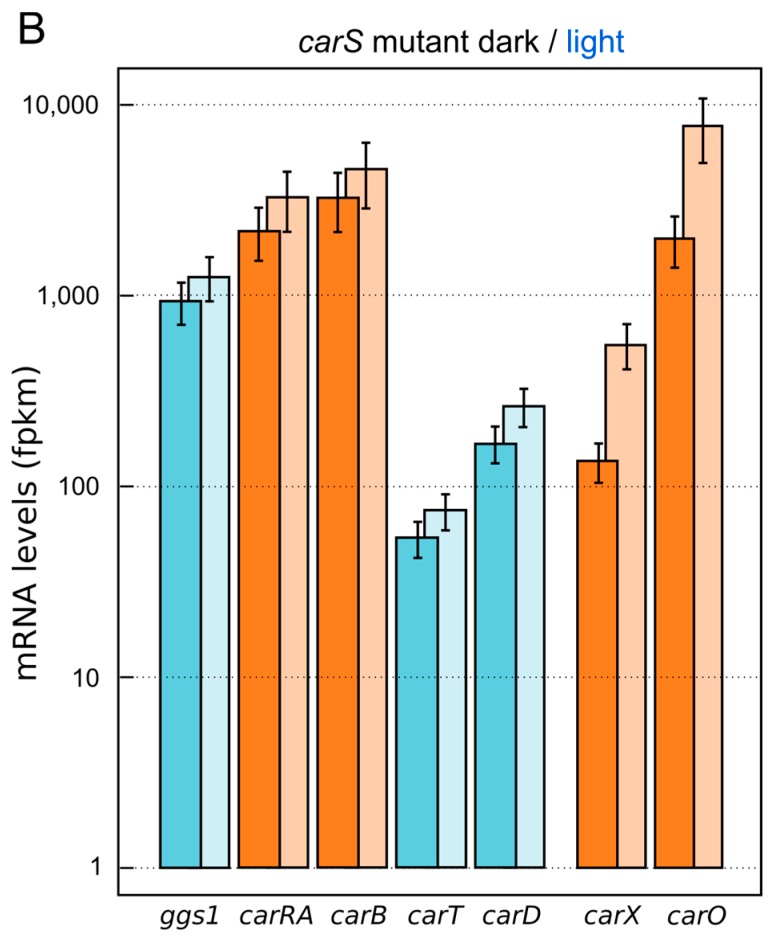
Effect of light and *carS* mutation on the expression levels for the *Fusarium* genes involved in carotenoid metabolism. The genes are grouped according to their functions: *ggs1*, *carRA*, *carB*, *carT,* and *carD* involved in NX biosynthesis and *carX* and *carO* involved in retinal and CarO rhodopsin production. Orange colors correspond to the genes organized as a cluster, and blue color to genes unlinked in the genome. Darker colors indicate cultures incubated in the dark and paler colors indicate cultures illuminated for one hour. (**A**) Above: wild-type strain IMI58289 of *F. fujikuroi* and its *carS* mutant SG39. Below: wild type strain 4287 of *F. oxysporum* (f. sp. *lycopersici*) and its *carS* mutant SX2; (**B**) Effect of light on the mRNA levels of the *carS* mutant SG39. RNA-seq data and culture conditions correspond to analyses already described [71]. In brief, all strains were cultured for three days in dark in minimal medium (DG medium for *F. fujikuroi* and DG with 3 g asparagine instead of NaNO_3_ for *F. oxysporum*). In the cases indicated, the cultures were exposed to one hour of illumination. RNA samples were sequenced with Illumina technology and the resulting data were analyzed with software tools for read mapping (Bowtie and TopHat), transcriptome assembly (Cuffmerge and Cufflinks), and differential gene expression analysis (Cuffdiff and CummeRbund).

The photoinduction of *Fusarium* carotenogenesis results from a rapid increase in the transcript levels of most of the structural genes. *Northern* blot experiments in *F. fujikuroi* showed similar induction kinetics for the four genes of the *car* cluster, *carRA*, *carB, carO* [59], and *carX* [38], as well as *carT* [42]. The transcripts reach maximal levels after one hour of exposure to light and decay afterwards, a down-regulating phenomenon known as photoadaptation. This photoinduction pattern has been confirmed by RT-qPCR approaches (Figure 2), and similar results have been obtained in *F. oxysporum* [68] and *F. verticillioides* [69]. The transcriptional photoresponse is similar to the one exhibited by the orthologous genes in *N. crassa* (reviewed by [70]), with the exception of the GGPP synthase gene *al-3*, that exhibits in this fungus a strong photoinduction while its *F. fujikuroi* ortholog *ggs1* is hardly affected by light [26]. Our recent RNA-seq data have revealed however a significant photoinduction of *ggs1* mRNA (Figure 3). A minor photoresponse was exhibited by the gene *carD* from *F. fujikuroi* [47], corroborated by the RNA-seq data (Figure 3), while no photoinduction was detected in the case of *ylo-1* from *N. crassa* [48], both genes encoding the ALDH enzymes responsible for the last reaction for NX production (Section 3). Therefore, in *F. fujikuroi*, the whole NX biosynthetic pathway is regulated by light.

The study of the transcriptional photoinduction of the structural genes for carotenogenesis in the mutants of the *wcoA* and *cryD* genes is consistent with different mechanisms of action for the encoded photoreceptors [65]. After illumination, the *wcoA* mutants exhibit photoinduction of carotenogenesis, but they accumulate carotenoids more slowly and their total levels do not reach those of the wild type. However, both the carotenoid content and the mRNA levels for the structural *car* genes are much lower in the dark in the *wcoA* mutants than in the wild type. Even more, the transcriptional photoinduction of the *car* genes is basically absent in the *wcoA* mutants, with mRNA levels after illumination lower than those of the wild type in the dark. Therefore, the induction of carotenogenesis by the alternative photoreceptor, presumably CryD, must be done through a transcription-independent mechanism. The molecular basis for such a mechanism, that could involve mRNA stability or translation and enzymatic activities or stabilities, remains to be elucidated.

The decay of the mRNA levels after photoinduction has been investigated in detail in *N. crassa*. In this fungus, the small flavin prohotoreceptor VIVID (VVD), its name due to the higher carotenoid levels of the *vvd* mutants under light, plays a key role in deactivating the WC photoreceptor system after illumination [70]. In contrast, the mutation of the VIVID ortholog in *F. fujikuroi*, called *vvdA*, results in a lower carotenoid accumulation in this fungus [72]. The kinetics of carotenogenesis in the *vvdA* mutants show a faster carotenoid accumulation immediately after light exposure, consistent with an attenuating function of VvdA on the photoactivated WcoA, but a slower accumulation after more prolonged growth under light, suggesting an up-regulating role during the second stage of carotenoid photoinduction [65].

## 5. Down-Regulation of Carotenoid Production: The *CarS* Gene

Standard mutagenesis protocols allow the identification of *Fusarium* mutants affected in the synthesis of carotenoids, easy to detect because of their changes in pigmentation. Such mutants are more easily identified on minimal medium, such as the DG minimal agar [73], than on richer media, e.g., Potato Dextrose Agar (PDA), which frequently results in the accumulation of other unrelated pigments which may mask the color of carotenoids. Carotenoid mutants usually identified by visual inspection are either albino in the light, as those lacking an early enzymatic step of the carotenoid pathway, or deeply pigmented in the dark, affected in a down-regulating function. The deep-orange mutants, exhibiting a light-independent carotenoid production and generically called *carS*, have been described in *F. fujikuroi* [22] and *F. oxysporum* [68]. The *carS* mutants have been investigated in detail in *F. fujikuroi*, where they accumulate an increasing amount of carotenoids with aging [55] and contain large amounts of mRNA for the structural genes, from *carRA* to *carD* [38,42,48,59] (Figure 3). This up-regulation results in an increase of carotenogenic enzymes, as deduced from the study of in vitro carotenogenesis in a cell-free system of a *carS* mutant compared to the wild type [74]. Because of their high carotenoid content, the effect of light is less apparent in the *carS* mutants, but expression studies have found a detectable photoinduction of the transcript levels for the structural genes. This has been found in *F. fujikuroi* [42] and in *F. oxysporum* [68], and it has been recently corroborated in *F. fujikuroi* in RNA-seq analyses (Figure 3).

**Figure 4:**
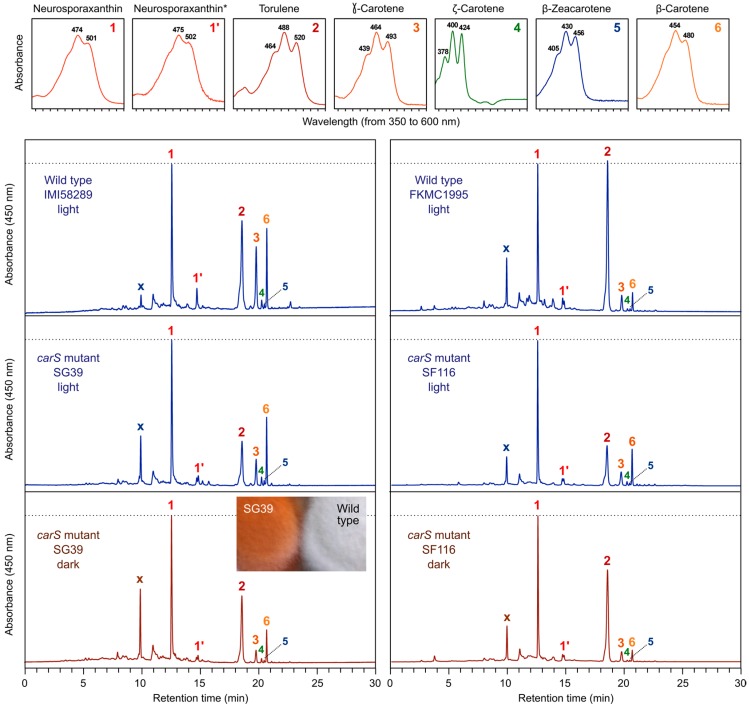
HPLC analysis of carotenoids produced by two different wild types of *F. fujikuroi* and the *carS* mutants SG39 [22] and SF116 [48]. The strains were grown for 7 days on DG minimal medium in the dark or under light (7 W m^−2^ white light). The method for HPLC separation has been described [75]. In brief, the samples were run in a reverse-phase C18 column with a binary-gradient elution (acetone : deionized water) at a flow rate of 1 mL/min. Detection was performed at 450 nm and online UV/Visible absorption spectra were acquired in the wavelength range 350–600 nm. The UV/Visible spectra of the identified carotenoids are shown above. The peak X corresponds to an unknown carotenoid with maximal absorbance at 480 nm, whose identity is under investigation. The peak 1’ is assigned as a neurosporaxanthin isomer. Phytofluene cannot be detected at 450 nm, but traces of this carotene were detected at 368 nm (data not shown). The inner picture shows the pigmentation of 7-day old colonies of the wild strain IMI58289 and the *carS* mutant SG39 grown on minimal DG medium in the dark.

The qualitative mixture of carotenoids accumulated by two *carS* mutants of *F. fujikuroi* obtained in different genetic backgrounds is similar to that of their corresponding wild types under light, with minor differences, mainly a lower torulene content in the *carS* strains (Figure 4). Because of the large accumulation of carotenoids in the dark, these mutants are also useful to investigate possible damaging effects of light on the carotenoids. The comparison of the chromatograms from dark or light-grown cultures in two *carS* mutants reveal again only minor changes, with a decrease in torulene and an increase in β-carotene in the samples from the illuminated cultures (Figure 4), which could be due to different sensitivities of these carotenes to light. Because of their intense pigmentation, the *carS* mutants have been useful tools to detect mutants with qualitative alterations in the carotenoid mixtures, e.g., making possible the identification of torulene accumulating mutants [22] or mutants with alterations in substrate recognition by the CarB desaturase [30]. The *carS* mutants have been also an optimal background to check the effect of possible carotenoid inhibitors on *Fusarium* carotenogenesis, revealing the differential efficiency of some cyclase inhibitors that were fully active in other species [55].

The gene *carS* was found to code for a protein of the RING finger (RF) family. The assignation was based in the identification of relevant mutations in all the *carS* mutants analyzed so far, the deep-pigmented phenotype resulting from the targeted mutation of the gene in *F. oxysporum* [68] and the complementation of the *carS* mutation in *F. fujikuroi* [76]. The predicted CarS protein has sequence similarity with the protein CrgA of *M. circinelloides*, whose mutation results in a similar carotenoid overproducing pattern in this species [77,78]. The degree of similarity between the CarS and CrgA proteins is not very high, as expected for two taxonomically distant fungi, but it covers the most relevant CrgA domains. These include two amino-terminal RF domains, one of them initially disregarded in CarS because of a wrong intron assignation in the *F. fujikuroi* genome annotation [71], and a LON protease domain [79]. However, CrgA has two glutamine-rich regions and a carboxy-terminal isoprenylation site, the first one underrepresented and the second absent in CarS. In other proteins, the RF domains interact with E3 ligase-type enzymes that mediate ubiquitylation of target proteins, frequently as a label for their degradation. The function of CrgA of *M. circinelloides* has received considerable attention, and the available information may provide some clues on the hypothetical way of action of CarS in *Fusarium*. At least one of the RF domains of CrgA is essential for its regulatory function in carotenogenesis, suggesting that this protein might function as an E3 ubiquitin ligase [78]. However, CrgA interacts with one of the three WC proteins of *M. circinelloides*, MCWC-1b, to trigger its degradation through an ubiquitylation-independent mechanism [80]. There are other similarities between CarS and CrgA functions. First, as found for the *carS* mutants in *Fusarium*, carotenoid biosynthesis maintains a detectable photoinduction in the *crgA* mutants of *M. circinelloides* [77]. Second, both CrgA and CarS are involved in other processes in addition to carotenogenesis, pointing to wider regulatory functions. This is supported by the alterations in growth and sporulation by the *crgA* mutants of *M. circinelloides* [81,82], and in gibberellin and bikaverin productions by the *carS* mutants of *F. fujikuroi* [83,84].

Interestingly, *carS* mutants have not been described in other *Fusarium* species and they could not be found in mutagenesis screenings in *F. verticillioides* (J. García-Martínez and V. Díaz-Sánchez, unpublished observations). However, the *carS* gene of *F. fujikuroi* is able to restore a basal carotenoid production upon introduction in a *carS* mutant of *F. oxysporum*, although not totally at the level of expression of the structural genes, indicating a functional conservation in both species [68].

## 6. Regulation by Light-Independent Factors

Nitrogen availability controls the production of different *Fusarium* secondary metabolites. Well-known examples are the productions of gibberellins, bikaverin, and fusarubin, induced by the absence of nitrogen through a complex regulatory network [85]. However, other metabolites, as fusarins and fusaric acid, exhibit an opposite regulatory pattern, with a higher production under excess of nitrogen [86]. Experiments with immobilized mycelia of *F. fujikuroi* incubated under low nitrogen conditions, to which excess nitrogen was subsequently added, revealed a higher synthesis of carotenoids under nitrogen starvation [87]. The negative effect of nitrogen was confirmed with the exchange of media between immobilized mycelia samples with different nitrogen contents, resulting in enhanced or reduced synthesis depending on the pass to low or high nitrogen conditions, respectively, indicating that the repressing effect is reversible.

In a different approach, the effect of nitrogen was studied in shake cultures of wild type and *carS* mutants. The production of carotenoids was higher in low nitrogen medium (low N/C ratio) than in high nitrogen medium (high N/C) in either of the tested strains [84]. A different set of experiments, in which cultures grown under an excess of nitrogen were transferred to a nitrogen-free solution, showed an increase of transcript levels of the structural genes *carRA* and *carB* after the transfer, which was accompanied by an enhanced accumulation of carotenoids. As found for photoinduction (see Section 3), the transcriptional increase was transitory, with maximal levels found in this case after more than 10 h following nitrogen deprivation.

The stimulatory effect of nitrogen starvation on *F. fujikuroi* carotenogenesis, either at mRNA or carotenoid levels, is additive with the one produced by light [84], indicating different activating mechanisms. The regulation of carotenogenesis by nitrogen has been also described in *N. crassa*, where the mRNA levels for the *carRA* and *carB* orthologs *al-1* and *al-2* increase considerably under nitrogen limitation compared to standard nitrogen conditions, either in the wild type or in blind *wc* mutants [88]. The regulation by nitrogen might involve control of expression at the level of chromatin structure, as indicated by the differences in histone methylation found for the genes of the *car* cluster in a mutant of the methyltransferase KMT6 in *Fusarium graminearum* [89]. In *F. fujikuroi*, the expression of several nitrogen-regulated clusters for secondary metabolite production correlate with H3K9 acetylation [90]. This suggests a similar mechanism in the regulation of the structural *car* genes, which is currently under investigation.

Other regulatory circuits may also influence the control of carotenoid biosynthesis. A major regulatory pathway is the one involving cAMP, produced by adenylate cyclase under the stimulation of a Gα unit from a heterotrimeric G protein. cAMP acts on other proteins, including protein kinases that phosphorylate target proteins to module their activity. The mutation of the adenylate cyclase gene *acyA* in *F. fujikuroi* results in higher levels of carotenoids in the dark but in a reduced photoinduction [91]. The mutation affects other phenotypes, as the growth pattern or the production of other secondary metabolites. Another study in the same species, that extended the analysis to the effect of mutations of two adenylyl cyclase-stimulating Gα subunits and two cAMP-dependent PKs, confirmed different alterations in secondary metabolism and development [92], but in this case the effect on carotenogenesis was not investigated. Carotenogenesis may also have a regulatory connection with sexual development, as suggested by the lower carotenoid photoinduction of the mutants of the MAT1-2-1 mating-type gene of *F. verticillioides* [69].

**Figure 5:**
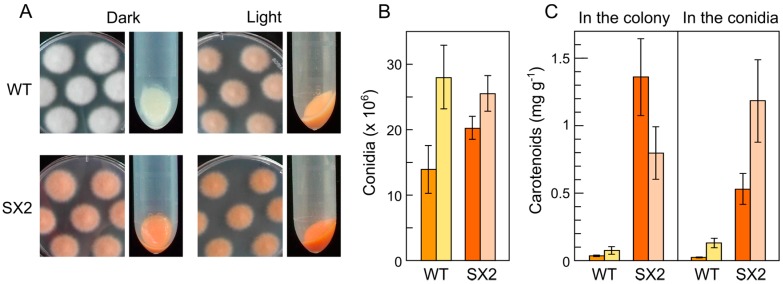
Conidiation and carotenoid biosynthesis in *F. oxysporum*. (**A**) Aspect of colonies of the wild type strain 4287 of *F. oxysporum* (f. sp. *lycopersici*) and its *carS* mutant SX2 grown on DG minimal medium at 30 °C for 7 days in the dark or under 5 w m^−2^ white light. Pictures on the right show the aspect of precipitated conidia obtained from the colonies as described [59]; (**B**) total amounts of conidia per Petri dish produced by the same strains under the indicated culture conditions; (**C**) total amounts of carotenoids accumulated in the mycelia and in the conidia mentioned above. The strains were grown in the dark (left darker bars) or under light (right paler bars). Data adapted from [93].

In *N. crassa*, besides the regulation by light, the synthesis of carotenoids is coupled to conidiation in a light-independent manner [70]. Actually, conidiation is stimulated by light and aerial growth in this fungus, leading to a massive conidia production that provides a typical orange pigmentation in slant cultures. Conidiation is less abundant in *Fusarium*, and the synthesis of carotenoids in the conidia has been usually disregarded. A comparison of the effect of light on the amount of carotenoids in mycelia and conidia from surface cultures in a wild type and a *carS* mutant of *F. oxysporum* showed the conservation in the conidia of the regulation by light and the deregulation by the *carS* mutation [93] (Figure 5). The low amount of carotenoids in wild type conidia in the dark indicates the lack of a developmental induction as the one described in *N. crassa*.

The cellular location of carotenoid biosynthesis is a regulatory aspect that has received little attention. Because of their hydrophobic nature, the carotenoids are assumed to interact with membranes, but their subcellular distribution in *Fusarium* is unknown. A biochemical approach based on the specific labeling of different terpenoids from ^14^C-labeled mevalonate allowed to establish that the carotenoids are produced in different cell compartments than those where the gibberellins and the sterols are synthesized [94]. Experiments to visualize the physical location of the enzymes for carotenoid biosynthesis in the fungal cells, based in their fusion to fluorescent proteins, are currently underway.

## 7. Conclusions and Future Prospects

Following the early studies in *F. aquaeductuum*, the fungus *F. fujikuroi,* and more recently also *F. oxysporum*, have become reference models in the research of fungal carotenogenesis and a wealth of information has been accumulated on the biochemistry and genetics of their NX production. All the genes and enzymes of the biosynthetic pathway are known and current research work is dedicated to elucidate the molecular mechanisms of the regulation. The available information on the control by light has revealed an unexpected complexity in relation to the model system *N. crassa*, in which the White Collar complex is the only transcriptional activator of the structural genes for neurosporaxanthin biosynthesis. At least a second photoreceptor, the DASH cryptochrome CryD, is also involved in the photoresponse in *Fusarium*. Our current research efforts are centered in a better understanding of the regulation by light and by the CarS protein, and their possible connections. Studies on the mechanism of action of CarS are focused in the alteration of its expression and in the identification of interacting proteins. Efforts are also centered in the identification of regulatory proteins able to bind the promoters of the structural *car* genes, which might include CarS targets. The regulation may include epigenetic mechanisms, as those related with chromatin structure, also under study. While the biochemistry of the carotenoid pathway has many similarities between different producing fungi, the regulatory mechanisms have been the object of a higher diversification, allowing refined adaptations to respond more efficiently to the different needs in their respective natural habitats. The studies on the regulatory processes governing carotenoid biosynthesis in *Fusarium* are a promising area of research, which predictably may lead to the discovery of novel molecular mechanisms.
